# Contrast-enhanced ultrasound detects changes in microvascular blood flow in adults with sickle cell disease

**DOI:** 10.1371/journal.pone.0218783

**Published:** 2019-07-05

**Authors:** Jonathan R. Lindner, Todd Belcik, Michael Widlansky, Leanne M. Harmann, Matthew S. Karafin, Nancy J. Wandersee, Maneka Puligandla, Donna Neuberg, Joel Linden, Joshua J. Field

**Affiliations:** 1 Knight Cardiovascular Institute, Oregon Health Sciences University, Portland, OR, United States of America; 2 Department of Medicine, Medical College of Wisconsin, Milwaukee, WI, United States of America; 3 Medical Sciences Institute, Versiti Wisconsin, Milwaukee, WI, United States of America; 4 Department of Biostatistics and Computational Biology, Dana-Farber Cancer Institute, Boston, MA, United States of America; 5 Division of Developmental Immunology, La Jolla Institute for Allergy and Immunology, La Jolla, CA, United States of America; 6 Department of Pharmacology, University of California San Diego, La Jolla, CA, United States of America; The University of Hong Kong, HONG KONG

## Abstract

In patients with sickle cell disease (SCD), poor outcome measures compromise the potential success of clinical trials. Contrast-enhanced ultrasound (CEUS) is a technique that can non-invasively quantify deep tissue microvascular blood flow. We tested the hypothesis that CEUS of forearm skeletal muscle could be used to: 1) assess microvascular abnormalities that occur during vaso-occlusive crisis; and 2) test new therapies for SCD that are targeted to improving the status of the microcirculation. We performed a prospective study, CEUS perfusion imaging of resting forearm muscle was performed in adults with SCD: 1) during and after a pain episode, and 2) before, during, and after a 24-hour infusion of the investigative agent, regadenoson, an adenosine A_2A_ agonist. CEUS destruction-replenishment time-intensity data were analyzed to measure microvascular blood flow, as well as its components, microvascular blood volume and flux rate. Serial CEUS measurements were obtained in 32 adults with SCD. For the studies during crisis, there was a 30% reduction in microvascular blood flow compared to steady-state (p = 0.031), a reduction that was largely due to microvascular flux rate. For the regadenoson group, a non-significant 25% increase in flux rate and 9% increase in microvascular blood flow compared to baseline were detected during infusion. In a study of adults with SCD, CEUS detected changes in microvascular blood flow associated with vaso-occlusive crises. No changes were found during an infusion of the adenosine A_2A_ agonist, regadenoson. This study provides preliminary evidence that CEUS could detect blood flow changes consistent with SCD physiology.

## Introduction

Sickle cell disease (SCD) is a group of inherited hemoglobinopathies that are characterized by red cell sickling. The result of mutations in the β-globin gene, the abnormal hemoglobin polymerizes in the deoxyhemoglobin state to create long fibers that cause red cells to change from bi-concave disks to sickle-shaped. Rigid, adhesive, and poorly able to traverse the microvasculature, sickle cells interact with white blood cells, platelets, and endothelial cells to obstruct capillaries and post-capillary venules, causing ischemia and tissue injury [1]. Vaso-occlusion in the bone marrow and lungs, the most commonly affected sites, produce episodes of pain and acute chest syndrome, which are leading causes of death [2–4]. These acute complications, as well as chronic end-organ damage, reduce the lifespan of patients with SCD to about 50 years [2]. Despite numerous insights into the underlying pathogenesis of SCD, this statistic has remained relatively constant for many years, likely because it has been challenging to identify new therapies [5–9]. Until L-glutamine became available in 2017, hydroxyurea was the only FDA-approved therapy for SCD [10,11]. Although the complex pathophysiology of the disease itself is the most significant barrier to the development of new therapies, another critical impediment is reliable outcome measures. Clinical outcome measures, such as frequency or duration of pain crises, or amount of opioids used, are subjective and vary from patient to patient [4,9,12]. Given that the underlying pathogenesis of SCD is microvascular occlusion, the measurement of microvascular blood flow (MBF), which should decrease in affected vessels, represents the most direct approach to assessing tissue status. Imaging MBF in the bone marrow, however, the main site of vaso-occlusion, is challenging and there is no blood flow measure that is commonly used in SCD clinical trials.

Contrast-enhanced ultrasound (CEUS) is a technique that uses standard ultrasound equipment to detect changes in MBF using microbubbles that are infused through a peripheral intravenous catheter. These commercially-available microbubbles are pure intravascular tracers that have a similar rheology as erythrocytes and oscillate in an ultrasound field thereby generating strong acoustic signals [13]. After a destructive pulse is delivered, the intravascular replenishment of the microbubbles provides data about MBF and its components, microvascular blood volume and flux velocity [14,15]. In this study, we extend findings by members of our research team that previously demonstrated the ability of CEUS to detect an increase in MBF between SCD patients treated with hydroxyurea compared to those without hydroxyurea [16]. We evaluated the ability of CEUS to detect changes in MBF in forearm skeletal muscle: 1) in SCD patients during vaso-occlusive crisis and after recovery, as a proof-of-concept to demonstrate the ability to detect dynamic perfusion abnormalities; and 2) sequentially in subjects undergoing an infusion of regadenoson, an adenosine A_2A_ agonist that has been investigated as a therapy for SCD. We hypothesized that CEUS-measured MBF would decrease during vaso-occlusive crisis and increase with regadenoson infusion.

## Methods

This study was approved by the Institutional Review Board at the Medical College of Wisconsin. Written consent was obtained from all subjects prior to participation and all study activities were performed according to the principles expressed in the Declaration of Helsinki. This study was registered at ClinicalTrials.gov (NCT01566890).

### Eligibility

Eligible subjects were adults with HbSS/HbSβ^0^-thalassemia and age 18 to 70 years, without known hypertension, left-right cardiac shunts, or previous sensitivities to ultrasound contrast agents. Subjects in the regadenoson and sickle cell control groups needed to be at least 2 weeks from their most recent hospital or emergency department visit, and at their baseline pain level. Subjects in the regadenoson group could not have a diagnosis of asthma.

### Study design

To examine the ability of CEUS to detect changes in MBF, we performed CEU studies in adult SCD patients: 1) during vaso-occlusive crisis as compared to steady-state, and 2) during an infusion of regadenoson (0, 6, and 24 hours) as compared to baseline, and also compared to SCD controls who did not receive regadenoson, but were similarly imaged at 0, 6, and 24 hours. For the crisis group, a vaso-occlusive crisis was defined as a hospitalization for pain for which no other explanation for the pain could be found^4^. Repeat CEUS examinations after hospitalization were performed at least one month after discharge, 2 weeks from an emergency department or hospital admission for any reason, and when the patient was at steady-state and reporting either no pain or baseline pain. For the regadenoson group, the study drug was administered at 1.44 mcg/kg/hr as a continuous infusion for 24 hours [9]. Prior to starting the infusion, a limited functional 2D echocardiogram was performed to exclude severe pulmonary hypertension, which was a previously documented safety concern for CEU that has since been refuted [17].

### CEU imaging

**Fig 1:**
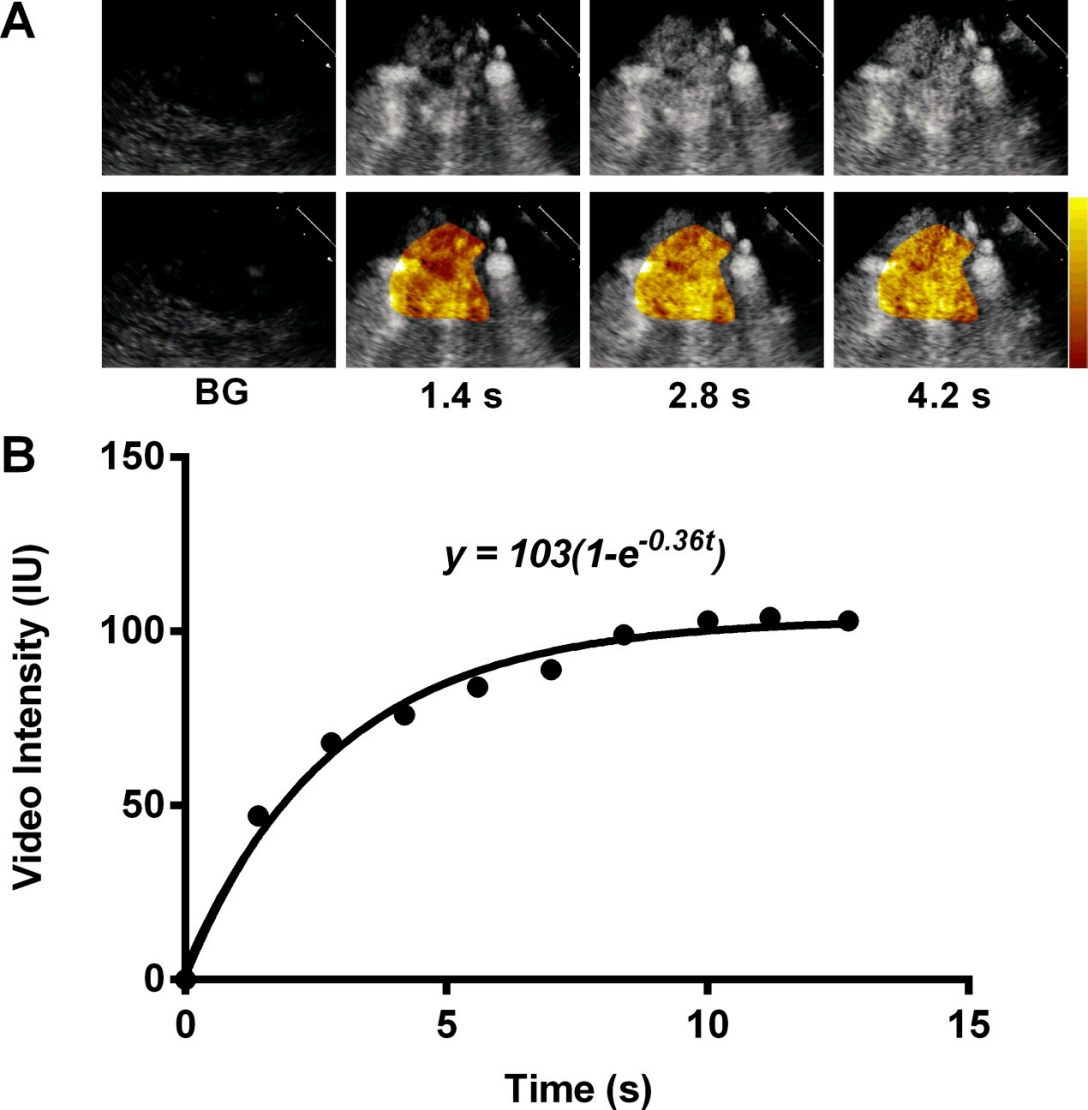
Contrast-enhanced ultrasound perfusion images and corresponding time intensity data from a subject at baseline. (A) Examples of raw images (top) and background-subtracted color coded images (bottom, scale at far right) within the quantitative region-of-interest demonstrating background images (BG) and contrast-enhancement at various time intervals after the destructive pulse sequence in forearm skeletal muscle. (B) Time intensity data derived from same subject.

Images were obtained as previously described [18] from the deep forearm flexor muscles (short-axis) after establishing a continuous infusion of microbubbles. The subject arm was scanned to locate the proximal forearm flexor muscles (flexor digitorum superficialis and flexor digitorum profundus) in the transverse plane. For the regadenoson group, the ultrasound transducer location was then marked with indelible ink to ensure identical anatomic location for each subsequent scan. These marks were not removed until all studies were complete. For the crisis group, upon location of the flexor muscles, a measurement was taken from the cubital fossa to the ultrasound transducer and recorded in centimeters on the ultrasound study sheet. This measurement was replicated at each subsequent exam. CEUS was performed for the first 3 crisis patients and the first 14 subjects undergoing regadenoson using lipid-shelled perfluorocarbon microbubbles (Definity, Lantheus Medical) within FDA-approved dosing guidelines.[19] However, because of pain episodes during infusion of Definity, which have been previously reported but occurred at a higher-than-expected rate [20], an alternative contrast agent composed of albumin-shelled perfluorocarbon microbubbles (Optison, GE Healthcare) was used for all studies thereafter [20]. Definity was administered by diluting 1 vial into 30 mL of saline and infusing at 1.5 ml/min. Optison was administered by diluting 1 vial into 30 ml saline and infusing at 1.5 ml/min. Imaging was started after allowing the microbubble concentration to reach a steady state (~2 min). We averaged the data from 3 separate perfusion runs. When examining the first, second, and third run; if there was no significant change in A-value, then the blood pool concentration was deemed stable since A-value simply reflected microvascular blood volume and blood contrast concentration. A programmable intravenous pump, Medfusion 3500 by Smith Medical, was used. All exams, whether the subjects were part of the regadenoson group, the control group, or the crisis group, and regardless of how many measurements were performed within an exam, were performed with a single contrast agent. Subjects in the regadenoson group each had two intravenous lines maintained, one for drug infusion and one for contrast infusion. Each subject ambulated only to and from the bathroom for the entire study duration (modified bed rest). Crisis subjects were also on modified bed rest at the time of the initial study due to pain and intravenous infusion pumps. For repeat exams, each subject was allowed to rest for about 15 minutes prior to study start. Across regadenoson subjects, the baseline, +6 hour, and +24-hour CEUS were performed at approximately the same time of day (± 2 hours). Contrast-specific power-modulation imaging (IE33, Philips Ultrasound) was performed at a transmission frequency of 1.7 MHz with a phased-array transducer at a mechanical index (MI) of 0.15. [14,21] Images were acquired for 15 seconds after a destructive (MI 1.0) 5-frame pulse sequence. Image analysis was performed by investigators who were not involved with image acquisition and were blinded to patient identity. Post-destructive time-intensity data were fit to the function y = A(1-e^βt^), where *y* is intensity at time *t*, *A* is the plateau intensity representing relative MBV, the rate constant β represents microvascular RBC flux rate, and the product of A and β represent MBF [22,23]. The 1-exp model was used as it is the only one that has been validated by microspheres and quantitative PET [22,24]. Further details on CEUS imaging is found in the supplemental methods. A sample CEUS with corresponding time intensity data from a subject at baseline are shown in Fig 1.

### Statistical analysis

Statistical analysis was performed using R version 3.5 and SAS version 9.4. Because skeletal muscle microvascular perfusion is influenced by factors that affect oxygen delivery [25,26], all values were normalized to hemoglobin which is known to vary in subjects with SCD. For comparison of crisis versus steady state condition CEUS values, a Wilcoxon signed-rank test was used. For the regadenoson subjects, a Wilcoxon signed-rank test on the ratios to baseline was used for each on-therapy value. There was significant variation at baseline in MBF, volume, and flow velocity in SCD subjects which was why we chose to analyze the change in ratios within a subject (Table A in S1 File). A power analysis demonstrates that for 20 regadenoson subjects, we have 90% power to detect a difference of ≥20%.

A mixed model was used to model the change in normalized A, β, and Axβ values over the 24-hour infusion, with measurements at baseline, 6 hours, and 24 hours. The model allowed for different slopes before and after the 6-hour mark. For the between-group comparison (regadenoson versus control), the 24-hour values were then modeled using ANCOVA with group (regadenoson or control) and baseline value as covariates. This approach assessed the effect of regadenoson on 24-hour values while controlling for the initial values. The primary hypothesis concerned MBF, and nominal p-values < 0.05 were considered statistically significant. Components of MBF were also tested, and nominal p-values were presented without adjustment for multiple comparisons.

## Results

### Demographics

**Fig 2:**
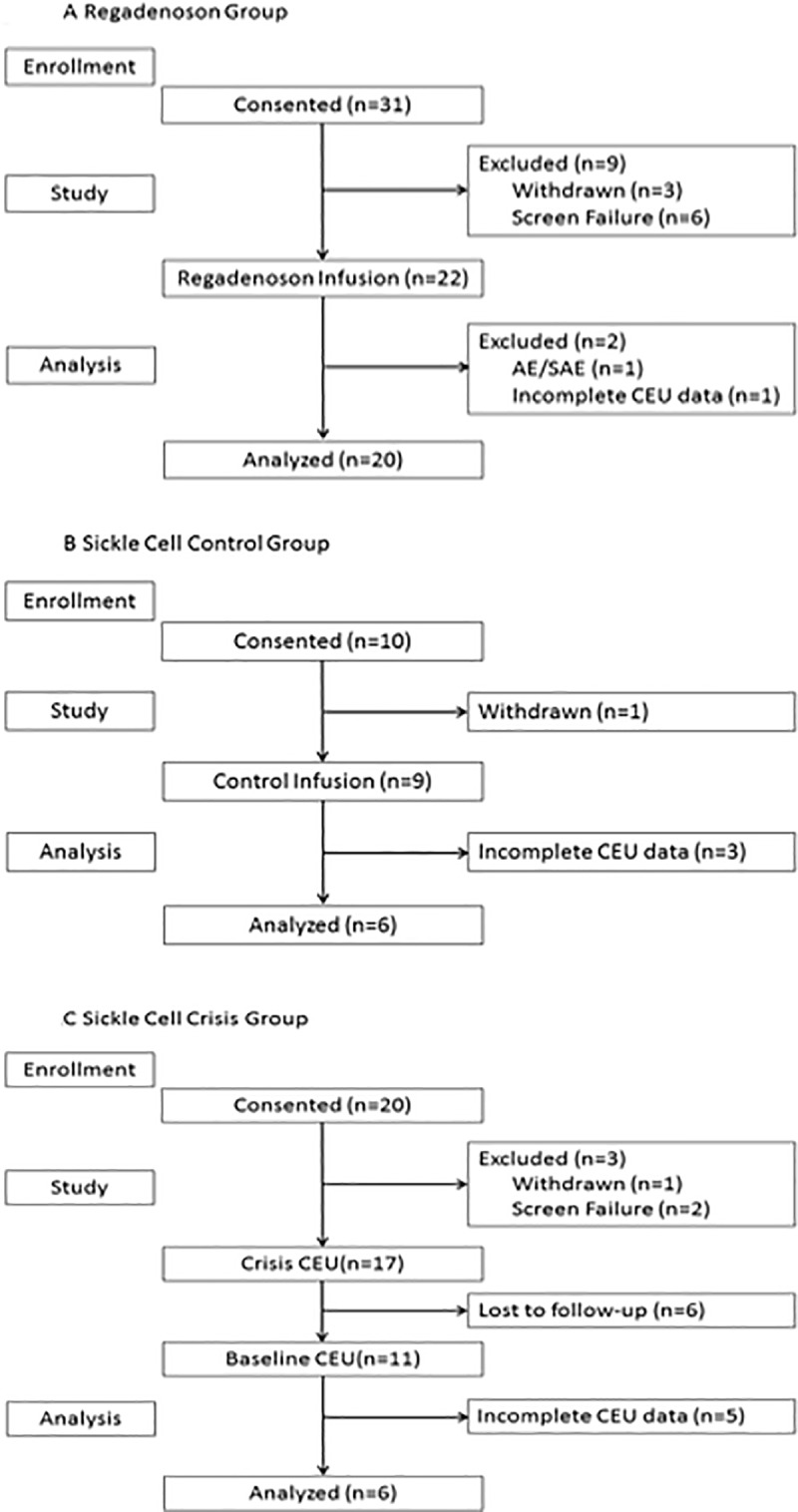
CONSORT flow diagram for the regadenoson. CONSORT flow diagram for the regadenoson (A), sickle cell control (B) and sickle cell CEUS (C) groups. Subjects were excluded if their data was incomplete.

Serial CEUS measurements were obtained in 32 subjects with SCD, 6 in the crisis group, 20 in the regadenoson group, and 6 in the SCD control group (Fig 2, Table 1). Of note, the median age of all SCD subjects was 26 years and 97% were treated with hydroxyurea.

**Table 1:** Patient demographics.

Characteristic	Regadenoson n = 20*	Sickle Cell Control n = 6*	Sickle Cell Crisis n = 6*
Male, # (%)	7 (35)	3 (50)	2 (33)
Age, median years (range)	25 (20–46)	35 (27–48)	26 (22–40)
BMI, median (range)	21 (18–31)	26 (0–29)	22 (17–28)
Hospitalizations, last 3 years, median (range)	4 (0–17)	4 (0–12)	4 (1–26)
ED visits, last 3 years, median (range)	2 (0–51)	4 (0–18)	3 (1–5)
Transfusions in last 3 years, median (range)	2 (0–129)	3 (0–25)	4 (0–9)
Hydroxyurea use, # (%)	20 (100)	5 (83)	6 (100)

*Shown are subjects with serial CEU. 2 regadenoson subjects, 3 sickle cell controls and 11 crisis subjects did not have complete data.

### Skeletal muscle perfusion abnormalities during crisis

**Fig 3:**
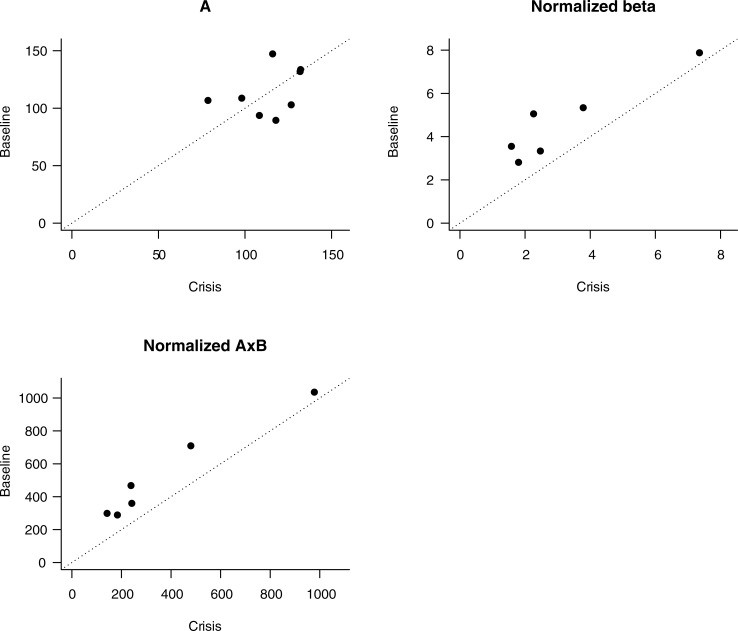
CEUS measurements normalized to hemoglobin in adults with SCD during vaso-occlusive crisis versus steady-state. Top left: Microvascular blood flow volume (A); top right: blood flow velocity normalized to hemoglobin (Normalized beta); bottom left, microvascular blood flow normalized to hemoglobin (Normalized AxB). Dots represent individual subjects. Dotted line indicates no change between baseline and crisis measurements. Dots above the line represent increases and dots below the line represent decreases between baseline and crisis measurements.

MBF, volume, and flow velocity was examined during a pain crisis and compared to steady-state values (Fig 3). During crisis, MBF was lower than steady-state, which was attributable primarily to a reduction in muscle microvascular flux rate (p = 0.031 for both). Microvascular blood volume was not significantly altered during crisis.

### The effects of regadenoson on MBF

**Fig 4:**
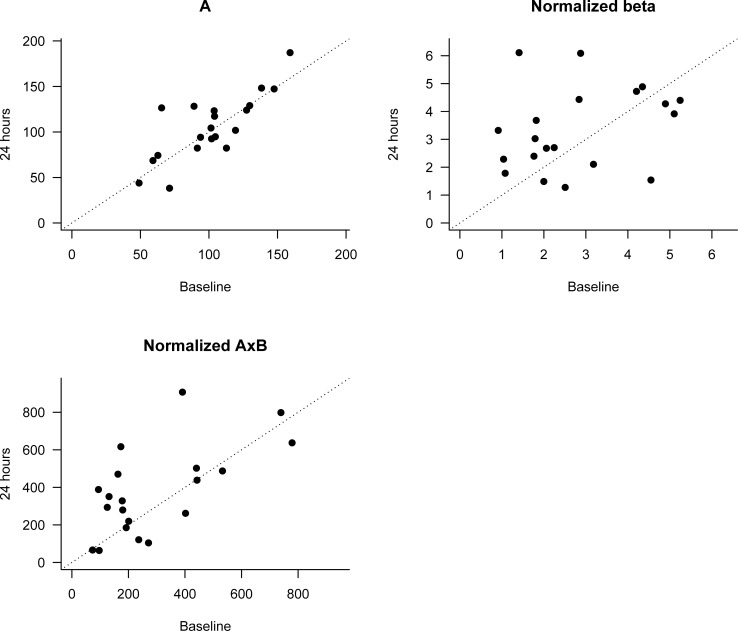
CEUS measurements normalized to hemoglobin in adults with SCD after 24-hour infusion of regadenoson versus baseline. Top left: Microvascular blood flow volume (A); top right: blood flow velocity normalized to hemoglobin (Normalized beta); bottom left, microvascular blood flow normalized to hemoglobin (Normalized AxB). Dotted line indicates no change between 24 hr and baseline measurements. Dots above the line represent increases and dots below the line represent decreases between 24 hr and baseline measurements.

After a 24-hour infusion of regadenoson, there was no significant effect of regadenoson on MBF in SCD subjects (Table 2, Fig 4). There were few differences in microvascular blood volume (A), suggesting the lack of recruitment of additional microvascular units. When CEU measurements at 24 hours were compared to baseline, 11/20 (55%) subjects showed an increase in MBF, with a median increase of 9% (p = 0.13). Thirteen of 20 (65%) of subjects demonstrated an increase in flux rate, with a median increase of 25% (p = 0.058). Despite these increases, neither MBF values nor flux rate achieved statistical significance; it is possible that increases in some subjects were offset by decreases in other subjects. There were no phenotypic differences noted in terms of responders (defined by an increase in MBF, as judged by AxB, in response to regadenoson, comparing 24 hours to baseline) vs. non-responders (Table B in S1 File).

**Table 2:** 24 hours/baseline in SCD subjects who were administered a 24-hour regadenoson infusion or control infusion.

	Regadenoson (n = 20)			Controls (n = 6)		
	A	β	Axβ	A	β	Axβ
**Min**	0.54	0.34	0.39	0.69	0.58	0.24
**1st quartile**	0.9	0.82	0.88	0.76	0.83	0.61
**Median**	1	1.25	1.09	0.77	0.94	0.86
**3rd quartile**	1.16	1.77	2.33	1.09	1.46	1.11
**Max**	1.93	4.33	4.12	1.33	1.77	2.82

Microvascular blood flow volume = A, flow velocity = β, microvascular blood flow = Axβ

To determine a change in normalized microvascular blood volume, flow velocity, and MBF over the 24-hour infusion with SCD patients, as well as between SCD patients treated with regadenoson and SCD controls who did not receive regadenoson, two models that accounted for repeat measures were explored: an ANCOVA and a mixed model (see Methods). The mixed model is a commonly used statistical technique that allows for the analysis of repeated measures. It is important to use this method because it accounts for the non-random nature of repeat measurements within an individual. Neither model yielded statistically significant results (Tables 3 and 4).

**Table 3:** ANCOVA model of changes in microvascular blood volume (A), flow velocity (β), and microvascular blood flow (Axβ) in SCD subjects administered a 24-hour regadenoson infusion or control infusion.

	A		β		Axβ	
	Estimate (95% CI)	P	Estimate (95% CI)	P	Estimate (95% CI)	P
**Intercept**	-0.56 (-25.1, 23.9)	0.96	1.39 (0.04, 2.8)	0.045	17.79 (-136.1, 171.7)	0.81
**Reg vs control**	11.85 (-12.0, 35.7)	0.31	1.11 (-0.2, 2.4)	0.09	160.91 (-14.8, 336.7)	0.071
**Baseline normalized Axβ**	0.93 (0.6, 1.3)	< 0.01	0.30 (-0.1, 0.7)	0.15	0.68 (0.29, 1.06)	<0.01

**Table 4:** Mixed model of changes in microvascular blood volume (A), flow velocity (β), and microvascular blood flow (Axβ) in SCD subjects administered a 24-hour regadenoson infusion or control infusion.

	A	β	Axβ
	Estimate (95% CI)	Estimate (95% CI)	Estimate (95% CI)
**Baseline**	37.05 (11.8, 62.3)	0.53 (-0.7, 1.7)	100.62 (-89.8, 291.0)
**6 hours**	39.30 (10.1, 68.5)	0.61 (-0.9, 2.1)	121.16 (-106.3, 348.6)
**24 hours**	35.96 (7.7, 64.2)	0.84 (-2.3, 4.0)	119.36 (-107.5, 346.2)

## Discussion

The primary aim of this study was to evaluate how CEUS can be applied to evaluate either pathogenic or therapeutic changes in tissue perfusion in patients with SCD. Using CEUS, we captured the expected decrease in MBF that should occur during a crisis as compared to steady state. In those patients who were administered a 24-hour infusion of regadenoson, there was an increase in MBF of 9%, an improvement that was due to flow velocity. Due to small sample size and high variability, these data did not achieve statistical significance, and therefore we cannot determine whether regadenoson is helpful for patients with SCD. However, our findings do show the potential applications of CEUS in SCD research.

There are no methods to measure blood flow that are routinely used in SCD. Ideally, a technique for assessing blood flow in SCD would: 1) be non-invasive, 2) measure MBF, as opposed to large vessel blood flow, 3) access a convenient and clinically-relevant site, and 4) be relatively inexpensive and use equipment that is widely available. To date, modalities that have been used to measure blood flow in SCD have not met these criteria. Notable examples include laser Doppler velocimetry, a technique that measures cutaneous blood flow, and slit-lamp microscopy, a technique that measures conjunctival blood flow [27–29]. In addition to the fact that both techniques require equipment that few institutions have, both examine vascular beds that may not be relevant to the bone marrow and have other drawbacks as well: skin vessels are sensitive to environmental changes and thus may not be an accurate reflection of the abnormalities of blood flow that are inherent to SCD and deep within organs; conjunctival vessels are branches off the cerebral vessels and may be affected by vasculopathy. Another technique that has been used in SCD is peripheral arterial tonometry, which non-invasively measures pressure changes in the fingertip. But, like laser Doppler velocimetry and slit-lamp microscopy, it requires specialized equipment and, additionally, it is measuring arterial tone, as opposed to MBF [30].

A key step to validate CEUS for use in SCD was to determine if it could identify changes in MBF that are an expected part of SCD pathogenesis. To accomplish this, we performed CEUS measures during a vaso-occlusive crisis as compared to steady-state. Indeed, we saw a significant reduction of MBF during crisis. Although expected, this result is an important proof-of-concept that helps to validate CEUS as a technique to measure MBF in patients with SCD. Additionally, it is important because the skeletal muscle is only a proxy for the vascular bed in which we are interested, the bone marrow, and, during crisis, there are likely many competing influences on microvascular perfusion. An imbalance between vasoconstrictor and vasodilator compounds, impaired rheology, microvascular thrombosis, and even anemia itself could all affect perfusion. To this last point, a recent study performed in patients with SCD under steady state conditions found that the true extent of their perfusion abnormality was only apparent after correcting for hemoglobin [16], which was why we corrected for anemia in this study as well.

Since measures of MBF would be an ideal outcome measure for clinical trials, we also wanted to evaluate the use of CEUS in the context of an investigational agent, in this case, regadenoson. We did not find evidence that regadenoson improved MBF, volume, or flow velocity in SCD subjects. Regadenoson is an adenosine A_2A_ agonist that is known as a vasodilator [31]. The basis for regadenoson’s FDA approval is its ability to dilate cardiac vessels and induce hyperemia during cardiac stress tests [32]. Our group has demonstrated that regadenoson also decreases activation of invariant NKT cells, which could decrease inflammation and reduce vaso-occlusion [33]. The fact that regadenoson did not improve MBF, volume, or flow velocity in SCD subjects is consistent with a recently-completed, multi-center trial of regadenoson for the treatment of pain crises in SCD, which was negative [9].

Although in this study no changes were found in MBF, volume, or flow velocity, the reason we chose to investigate CEUS was because it has been shown in other disease states to provide insights into the determinants of tissue perfusion [14,15]. Changes in the A-value reflect MBV. Alternatively, changes in microvascular flux rate (β) can occur from either changes in rheology at the capillary level or changes in the tone of resistance arterioles that govern pre-capillary pressure [13]. When testing new therapies that are designed to improve microvascular tone or erythrocyte rheology, CEUS could provide important proof-of-mechanism information.

As with any study, ours had limitations. The most significant limitation was our small sample size. There is a high degree of inter-individual variability in MBF that is part of normal physiology. Regardless of what technique is used to measure MBF, this will be a limitation that will be affected by small sample sizes. Potentially, CEUS could miss small differences that may be clinically meaningful in a therapeutic trial. The performance of repeat studies and analyzing fold changes within an individual is likely the best approach to overcome this limitation.

In this study, we evaluated CEUS, a technique that measures MBF in the skeletal forearm muscle. Although skeletal muscle is a good proxy for the vascular bed of interest, it is still a proxy. Nevertheless, there are several attributes of this technique that make it appealing for SCD trials. CEUS is non-invasive, uses common ultrasound equipment, and requires minimal training. Although we did not find differences in MBF associated with regadenoson, this study provides preliminary evidence that CEUS could be used as an outcome measure for other therapeutic trials.

## Supporting information

S1 File CEUS imaging methods and Table A and B.
(DOC)
