# Supplementary material for: Contrast-enhanced ultrasound detects changes in microvascular blood flow in adults with sickle cell disease
Source: PLoS One. 2019 Jul 5;14(7):e0218783. doi: 10.1371/journal.pone.0218783 (PMC6611596; doi:10.1371/journal.pone.0218783)
Supplement: S1 File — CEUS imaging methods and Table A and B. (DOC) [file pone.0218783.s001.doc]

**SUPPLEMENTAL METHODS**

*CEU Imaging*

Microvascular replenishment kinetics were evaluated from the progressive prolongation of high-power destructive pulse sequences^22^. The proximal forearm flexor muscles (flexor digitorum superficialis and pro-fundus) were imaged in the transverse plane ≈1/3 the distance to the wrist using a phased-array transducer interfaced with an ultrasound system (iE33, Philips Ultrasound). Power modulation imaging was performed using a broad-band pulse centered at 2.0 MHz at a mechanical index of 1.0, with the acoustic focus placed at the mid-muscle level. One vial of lipid-shelled octafluoropropane microbubbles (Definity, Lantheus Medical Imaging) or albumin-shelled perfluorocarbon microbubbles (Optison, GE Healthcare) were diluted to a total volume of 30 mL in 0.9% saline for a final concentration of 5×10^8^mL.^22^

The microbubble suspension was infused IV at 1.5 mL/min. Imaging was started after allowing microbubble concentration to come to steady state (~2 min). A short (2- to 3-s) continuous imaging sequence was followed by acquisition of intermittent frames obtained at incrementally increasing time intervals from every 1 to 15 cardiac cycles at end diastole by gating acquisition to the electrocardiographic R-wave. Several frames were acquired for each pulsing interval. The total time of acquisition was ≈3 to 4 minutes. This process was repeated 3 times over ≤15 minutes. Progressive prolongation of the pulsing interval guaranteed exposure of microbubbles to ultrasound within the entire span of the muscle circulation and allowed quantification of perfusion. Several continuous imaging frames were averaged and digitally subtracted from averaged frames at each pulsing interval. Time versus background-subtracted video intensity data from the muscle were fit to the function y=A(1-eβt), where y is intensity at time t, A is the plateau intensity representing relative microvascular blood volume, and the rate constant β is the microvascular flux rate.^22^ Microvascular blood flow was quantified by the product of A and β.^22^ The 1-exp model was used as it is the only one that has been validated by microspheres and quantitative PET^22,24^. A sigmoid curve happens when too aggressive of a burst is used where the volume of destruction is much larger than the volume of the imaging plane (resulting in a delay until replenishment happens).

**SUPPLEMENTAL RESULTS**

There was significant variation at baseline in MBF, volume, and flow velocity in SCD subjects which was why we chose to analyze the change in ratios within a subject (**Table A**).

**Table A.** Variation in CEUS at baseline

|  | **Mean** | **Median** | **SD** |
| --- | --- | --- | --- |
| Normalized beta |  | | |
| Control  Regadenoson | 2.2 | 2.3 | 1.1 |
|  | 2.8 | 2.2 | 1.4 |
| Normalized AxB |  | | |
| Control  Regadenoson | 185.1 | 136.1 | 153.8 |
|  | 288.0 | 201.0 | 203.1 |
| Normalized A |  | | |
| Control  Regadenoson | 64.1 | 57.2 | 23.1 |
|  | 101.4 | 101.9 | 29.4 |

Comparison of 24 hour to baseline changes in the normalized plateau (A) measurement between Definity (n=14, median change -0.01, change range -17.6 – 61.0) and Optison (n=6, median change 2.1, change range -33.0 – 13.1) did not reveal a significant difference between contrast agents (p=0.49).

There were no phenotypic differences noted in terms of responders (defined by an increase in MBF, as judged by AxB, in response to regadenoson, comparing 24 hours to baseline) vs. non-responders (**Table B**).

**Table B. Phenotypic** Comparison of Responders vs Nonresponders

|  | **Responder* (N=11)** | **Nonresponder (N=9)** | **P-value** |
| --- | --- | --- | --- |
| Male, n (%) | 3 (27) | 4 (44) | 0.6 |
| Age, yrs, median (range) | 25.0 (23.0-43.0) | 25.0 (20.0-46.0) | 0.9 |
| BMI, median (IQR) | 22.8 (17.9-31.1) | 20.0 (18.7-28.1) | 0.6 |
| Cardiac Ejection Fraction, median (IQR) | 61.3 (41.6-74.6) | 58 (52.5-67.9) | 0.5 |
| Reported pain  Baseline  At 24 hours  Change in pain | 0.1 (0-5.3)  0.0 (0.0-5.2)  0.0 (-1.3-4.5) | 0.0 (0.0-5.2)  0.0 (0.0-4.0)  0.0 (-4.0-4.6) | 0.5  0.5  0.6 |
| Hospital Utilization, median (IQR)  ED visits  Hospital Admissions | 1.0 (0-51.0)  4.0 (0-17.0) | 4.0 (0-16.0)  4.0 (0-13.0) | 0.6  0.9 |
| Comorbidities,n (% yes)  Avascular necrosis  Stroke  Pulmonary Hypertension  Acute chest syndrome  Daily pain  Iron overload | 1 (9.0)  1 (9.0)  1 (9.0)  2 (18.2)  4 (36.3)  2 (18.2) | 3 (33.3)  1 (11.1)  0 (0.0)  1 (11.1)  3 (33.3)  3 (33.3) | 0.3  1.0  1.0  1.0  1.0  0.6 |
| Medication use, n (% yes)  Blood pressure  Diuretics  Anticoagulants  Opioids | 2 (18.2)  0  1 (9.0)  11 (100) | 1 (11.1)  0  0 (0.0)  9 (100) | 1.0  NA  1.0  1.0 |
| Reagent use, n (% yes)  Definity  Optison | 9 (82)  2 (18) | 5 (55)  4 (45) | 0.3 |

*Responder=Increase in MBF (as judged by normalized AxB) in response to regadenoson (24 hr vs. baseline). Pulmonary hypertension is defined by a tricuspid regurgitant jet velocity >3 m/sec. Iron overload is defined by a ferritin >1500 or a value of 3 mg/gm of iron in liver by MRI or biopsy.
